# Supplementary material for: Structural models for spreading and scaling digital health initiatives: A scoping review protocol
Source: PLoS One. 2026 Feb 26;21(2):e0336730. doi: 10.1371/journal.pone.0336730 (PMC12944785; doi:10.1371/journal.pone.0336730)
Supplement: Appendix 1 — (DOCX) [file pone.0336730.s001.docx]

**Appendix 1: Medline Search Strategy**

Ovid MEDLINE(R) ALL <1946 to present>

| **#** | **Searches** |
| --- | --- |
| 1 | Diffusion of Innovation/ |
| 2 | Organizational Innovation/ |
| 3 | Implementation Science/ |
| 4 | (scale*-up? or scaleup? or scale*-out? or scaleout? or scaling-out? or scaling-up?).tw,kf. |
| 5 | ((scale* or scalab* or scaling) adj3 (science or infrastructure or efficien* or inefficien*)).tw,kf. |
| 6 | ((scale* or scalab* or scaling or up-scale* or upscale* or up-scaling or upscaling or roll*-out* or rollout* or spread? or spreading or widespread) adj5 (innovation? or intervention? or technolog* or practice* or practise* or care or initiative* or framework* or model* or program* or product? or therap* or service* or strateg* or change? or process or processes)).tw,kf. |
| 7 | (scale* adj3 spread*).tw,kf. |
| 8 | ((bring* or brought or taking or take* or increas* or going or implement* or econom*) adj5 scal* adj5 (innovation? or intervention? or technolog* or practice* or practise* or care or initiative* or framework* or model* or program* or product? or therap* or service* or strateg* or change? or process or processes)).tw,kf. |
| 9 | ((vertical* or horizontal* or diversif* or spontaneous*) adj3 (scala* or scale* or scaling or spread* or integrat*)).tw,kf. |
| 10 | ((enfranchis* or franchis*) adj3 (social* or clinical* or health*)).tw,kf. |
| 11 | (anchor adj5 network*).tw,kf. |
| 12 | (((diffus* or disseminat*) adj3 innovat*) or implementation science).tw,kf. |
| 13 | ((hub or hubs) adj3 (spoke or spokes)).tw,kf. |
| 14 | ((hub or hubs or spoke or spokes) adj4 (clinic? or model* or approach* or framework* or hospital* or network* or organis* or organiz* or site* or system*)).tw,kf. |
| 15 | ((multi-site* or multisite* or multiple site*) adj4 (center? or centre? or clinic? or facility or facilities)).tw,kf. |
| 16 | action research.tw,kf. |
| 17 | or/1-16 [***CONCEPT 1 - SCALING UP***] |
| 18 | exp Telemedicine/ |
| 19 | Digital Health/ |
| 20 | internet-based intervention/ |
| 21 | Videoconferencing/ |
| 22 | Webcasts as Topic/ |
| 23 | Cell Phone/ |
| 24 | Smartphone/ |
| 25 | Text Messaging/ |
| 26 | Wearable Electronic Devices/ |
| 27 | Electronic Health Records/ |
| 28 | Health Information Exchange/ |
| 29 | exp Artificial Intelligence/ |
| 30 | (digital-first or digital first).tw,kf. |
| 31 | ((cyber or digital or remote* or distance* or online or tele or virtual*) adj2 (care or collaborat* or consult* or conferenc* or counsel* or educat* or diagnos* or health or guide* or diagnos* or learn* or medical* or medicine or mentor* or monitor* or platform* or presence* or screen* or therap* or transmi*)).tw,kf. |
| 32 | ((cyber or digital or distance* or electronic* or tele or remote* or video* or virtual* or sms or phone* or cellphone* or cell-phone* or cell phone* or smartphone* or smart-phone* or smart phone* or internet or web or web-based or web based or webcast* or web-cast* or web cast* or telephone* or text messag* or texting or 2wT or asynchronous messag* or synchronous messag* or iphone* or Android* or app or apps or ((digital or mobile or phone* or smartphone*) adj2 (application* or tool*)) or Instagram or Snapchat or Facetime or GMeet* or Google Hangout* or Skype or Zoom or Web-ex or WebEx or WhatsApp or Bluejeans or Facebook or Messenger or Microsoft Teams or MS Teams or e-mail* or email* or e-chat or echat or "social media" or "text message*" or hotline* or helpline* or help line* or call center* or call centre* or "answering machine*" or iPad* or tablet* or voicemail* or voice mail*) adj3 (communicat* or engag* or discuss* or care or interact* or clinical guidance)).tw,kf. |
| 33 | ((cyber or digital or distance* or electronic* or tele or remote* or video* or virtual* or sms or phone* or cellphone* or cell-phone* or cell phone* or smartphone* or smart-phone* or smart phone* or smart device* or internet or web or web-based or web based or webcast* or web-cast* or web cast* or telephone* or text messag* or texting or 2wT or asynchronous messag* or synchronous messag* or iphone* or Android* or app or apps or ((digital or mobile or phone* or smartphone*) adj2 (application* or tool*)) or Instagram or Snapchat or Facetime or GMeet* or Google Hangout* or Skype or Zoom or Web-ex or WebEx or WhatsApp or Bluejeans or Facebook or Messenger or Microsoft Teams or MS Teams or e-mail* or email* or e-chat or echat or "social media" or "text message*" or hotline* or helpline* or help line* or call center* or call centre* or "answering machine*" or iPad* or tablet* or voicemail* or voice mail*) adj3 (appointment* or consult* or support* or diagnos* or exam* or followup* or follow-up* or meet or meeting* or health or doctor* or physician* or primary care or clinic or clinics or clinician* or nurs* or psycholog* or therap* or intervention* or delivery or refer* or session* or visit* or refer*)).tw,kf. |
| 34 | ((cyber or digital or electronic or online or remote* or tele or patient* or video* or virtual or web or web-based) adj2 portal*).tw,kf. |
| 35 | ((cyber or digital or electronic or online or remote* or tele or video* or virtual or web or web-based) adj3 (health or healthcare or medical or medicine)).tw,kf. |
| 36 | (cybertherap* or cyber-therap* or teleassist* or "tele‐assist*" or teleaudiolog* or "tele‐audiolog*" or telebased or "tele‐based" or telecancer or "tele‐cancer" or telecardiolog* or "tele‐cardiolog*" or telecare or "tele-care" or telecollaborat* or "tele-collaborat*" or telecommunicat* or "tele-communicat*" or teleconsult* or "tele-consult*" or teleconferenc* or "tele-conferenc*" or telecounsel* or "tele‐counsel*" or teleconference* or "tele-conference*" or telecounsel* or "tele‐counsel*" or teledental or "tele‐dental" or telederm* or "tele‐derm*" or telediagnos* or "tele‐diagnos*" or teledialysis or "tele‐dialysis" or teleecho* or "tele‐echo*" or teleeducat* or "tele-educat*" or teleemerg* or "tele‐emerg*" or teleepileps* or "tele-epileps*" or telefollow* or "tele‐follow*" or teleguidance or "tele‐guidance" or telehealth or "tele‐health*" or telehome* or "tele‐home*" or teleguide* or "tele-guide*" or teleICU or "tele‐ICU" or teleintervention* or "tele‐intervention*" or telelearn* or "tele-learn*" or telemanag* or "tele‐manag*" or telemed* or "tele-med*" or telemental* or "tele‐mental*" or telementor* or "tele-mentor*" or telemonitor* or "tele-monitor*" or teleneurol* or "tele-neurol*" or telenurs* or "tele‐nurs*" or teleoncolo* or "tele‐oncolo*" or teleopthalm* or "tele-opthalm*" or telepalliat* or "tele‐palliat*" or telepatholog* or "tele-patholog*" or telepediatric* or "tele-pediatric*" or telepresence* or "tele-presence*" or teleprocedu* or "tele‐procedu*" or telepsych* or "tele‐psych*" or teleradiol* or "tele-radiol*" or telerefer* or "tele‐refer*" or telerehab* or "tele-rehab*" or telerobotic* or "tele-robotic*" or telescreen* or "tele-screen*" or telesurger* or "tele‐surger*" or telesurgic* or "tele-surgic*" or teletherap* or "tele‐therap*" or teletreat* or "tele‐treat*" or teletriage or "tele‐triage*" or teletransmi* or "tele-transmi*").tw,kf. |
| 37 | (ecare or "e‐care" or eclinic or e-clinic* or econsult* or "e‐consult*" or ediagnosis* or "e‐diagnos*" or emedicine or "e‐medicine" or enurse* or "e‐nurse*" or enursing or "e‐nursing" or edoctor* or "e-doctor*" or ephysician* or "e‐physician*" or epsych* or "e‐psych*" or etherap* or "e‐therap*" or ehealth or "e-health" or evisit* or "e-visit*" or mhealth or "m‐health").tw,kf. |
| 38 | ((implant* sensor* or body sensor*) adj4 (diagnost* or monitor* or report*)).tw,kf. |
| 39 | ((mobile adj3 monitor*) or wearable*).tw,kf. |
| 40 | (electronic health record* or EHR* or ((health or medical) adj2 exchange)).tw,kf. |
| 41 | (AI or "artificial* intelligen*" or AIVI or "classification algorithm*" or "computer heuristic*" or "convolutional network*" or DALL-E or "decision support system*" or "decision tree" or DeepAI or "deep learning" or "data science" or "feature detection" or "genAI" or "generative pre-trained transformer" or "generative pretrained transformer" or Invideo or "language learning model*" or "large language model*" or LLM* or "learning algorithm*" or "machine learning" or (Markov adj3 model*) or Midjourney or ((multifactor* or multicriteria) adj3 ("decision analysis" or "decision making")) or "natural language process*" or NLP* or "nearest neighbo*" or "neural network*" or "outlier detection" or "pattern recognition" or Perplexity or "probability tree" or "random forest" or "representation learning" or Runway AI or Runway Gen-1 or "Stable Diffusion" or "support vector machine*" or "transfer learning" or "Bing chat" or ChatGPT* or "Chat GPT*" or chatbot* or chat-bot* or "Google* Bard" or "Google* Gemini" or "IBM Watson" or "Microsoft* Bing" or "Microsoft* Copilot" or OpenAI or "Open AI" or PathAI or "Path AI" or DeepSeek or Grok).tw,kf. |
| 42 | or/18-41 [***CONCEPT 2 - DIGITAL HEALTH***] |
| 43 | "Delivery of Health Care"/ |
| 44 | "Delivery of Health Care, Integrated"/ |
| 45 | Health Services Accessibility/ |
| 46 | Access to Primary Care/ |
| 47 | models, organizational/ |
| 48 | Models, Nursing/ |
| 49 | Models, Theoretical/ |
| 50 | "organization and administration"/ |
| 51 | ((deliver* or implement* or integrat*) adj3 (care or health* or service* or system*)).tw,kf. |
| 52 | (access* adj3 (care or health* or service* or system*)).tw,kf. |
| 53 | (care adj2 model*).tw,kf. |
| 54 | or/43-53 [***CONCEPT 3 - CARE DELIVERY AND MODELS***] |
| 55 | (exp Animals/ or exp Animal Experimentation/ or Disease Models, Animal/ or exp Models, Animal/ or exp Models, Molecular/ or exp Plants/ or exp Forests/ or exp Cells/ or exp Cell Physiological Phenomena/ or exp Proteins/) not Humans/ |
| 56 | ((animal or animals or bird or birds or fish or fishes or canine* or dog or dogs or feline or hamster* or lamb or lambs or mice or monkey or monkeys or murine or pig or pigs or piglet* or porcine or primate* or rabbit* or rat or rats or rodent* or sheep* or veterinar* or plant or plants or forest or forests or cellular or proteins or (molecular adj1 model*)) not (human* or patient*)).tw,kf,jw. |
| 57 | 55 or 56 [animal, plant, molecular studies] |
| 58 | 17 and 42 and 54 [***CONCEPTS 1 and 2 and 3 with only care delivery/models***] |
| 59 | 58 not 57 [***CONCEPTS 1 and 2 and 3 with only care delivery/models***, excl animals***] |
| 60 | limit 59 to yr="2003 -Current" |
