# Supplementary material for: Structural models for spreading and scaling digital health initiatives: A scoping review protocol
Source: PLoS One. 2026 Feb 26;21(2):e0336730. doi: 10.1371/journal.pone.0336730 (PMC12944785; doi:10.1371/journal.pone.0336730)
Supplement: Attachment — Submitted filename: Response to Reviewers-Jan20,2026.docx [file pone.0336730.s004.docx]

**Response to Reviewers**

Thank you for reviewing this protocol and providing these useful suggestions. We have made all suggested changes.

1. **We note your current Data Availability statement: "N/A - No results are reported-
   Given the broad approach of extracting text whose relevance may not be determined until the analysis stage, the data extraction will not be made publicly available. However, other researchers are welcome to contact the team to receive that data, with limitations clearly outlined. Codebooks and any guiding lists of known models of spread and scale and definitions will be made publicly available."

   Please note that we recommend the following Data Availability Statement for Study Protocols:
   "No datasets were generated or analyzed during the current study. All relevant data from this study will be made available upon study completion.**

This change has been made, and the Data Availability Statement now reads:

*No datasets were generated or analyzed during the current study. All relevant data from this study will be made available upon study completion.*
